# A qualitative study of a social and emotional well-being service for a remote Indigenous Australian community: implications for access, effectiveness, and sustainability

**DOI:** 10.1186/1472-6963-13-80

**Published:** 2013-03-04

**Authors:** Timothy A Carey

**Affiliations:** 1 Centre for Remote Health, a joint Centre of Flinders University and Charles Darwin University, PO Box 4066, Alice Springs, NT, 0871, Australia; 2 NT Department of Health and Families, Central Australian Mental Health Service, Alice Springs, Australia; 3 Centre of Research Excellence in Rural and Remote Primary Health Care funded by the Australian Primary Health Care Research Institute, Alice Springs, Australia

**Keywords:** Social and emotional wellbeing, Effectiveness, Sustainability, Indigenous Australians, Rural, Remote

## Abstract

**Background:**

People living in rural and remote Australia experience increased mental health problems compared with metropolitan Australians. Moreover, Indigenous Australians are twice as likely as non Indigenous Australians to report high or very high levels of mental health problems. It is imperative, therefore, that effective and sustainable social and emotional wellbeing services (Indigenous Australians prefer the term “social and emotional wellbeing” to “mental health”) are developed for Indigenous Australians living in remote communities. In response to significant and serious events such as suicides and relationship violence in a remote Indigenous community, a social and emotional wellbeing service (SEWBS) was developed. After the service had been running for over three years, an independent evaluation was initiated by the local health board. The aim of the evaluation was to explore the impact of SEWBS, including issues of effectiveness and sustainability, from the experiences of people involved in the development and delivery of the service.

**Methods:**

Purposive sampling was used to recruit 21 people with different involvement in the service such as service providers, service participants, and referrers. These people were interviewed and their interviews were transcribed. Interpretative Phenomenological Analysis (IPA) was used to analyse the interview transcripts to identify superordinate themes and subthemes in the data.

**Results:**

Two superordinate themes and nine subthemes were developed from the interview transcripts. The first superordinate theme was called “The Big Picture” and it had the sub themes: getting started; organizational factors; funding; the future, and; operational problems. The second superordinate theme was called “On the Ground” and it had the subthemes: personal struggles; program activities; measuring outcomes, and; results.

**Conclusions:**

While the evaluation indicated that the service had been experienced as an effective local response to serious problems, recommendations and directions for future research and development emerged that were more broadly applicable. Issues such as appropriate staffing, localising decision making, identifying priorities and how they will be evaluated, and developing flexibility in terms of job descriptions and qualifications are highlighted.

## Background

Indigenous Australians are twice as likely as non Indigenous Australians to report high or very high levels of psychological distress [1,2]. In fact, mental health problems are the second largest (15%) contributor to total disease burden for Indigenous Australians with only cardiovascular disease making a greater contribution [3]. While these findings are concerning enough, they become even more serious when the links to physical ill health are considered. Kelly et al. report that serious psychological distress is an independent predictor of physical illness and mortality risk [4]. High levels of psychological distress, for example, are associated with reduced life expectancy and a greater incidence and prevalence of disease. Despite mental health problems causing significant disability and disruption to daily functioning, less than one third of Indigenous people access any form of mental health service [5]; particularly those in rural and remote communities [6].

Western notions of mental health do not translate easily into concepts that are meaningful for Indigenous Australians. It has been suggested that the term “social and emotional wellbeing” is a more appropriate way of capturing the holistic sense of health and connectedness that is important to Indigenous Australians [4]. Indigenous wellbeing is also firmly culturally based [7]. For interventions to be effective, therefore, they need to be culturally attuned to the people to which they are being offered. The cultural sensitivity of interventions is especially important with Indigenous Australians and context is a critical consideration [8] given that they do not represent a homogenous culture. To ensure that interventions are appropriate and meaningful for local cultural groups, therefore, programs should be developed within individual communities [9]. Local involvement in health promotion and the ownership and definition of problems and solutions by community members, for example, were identified in a review of published studies as factors that are critical to the success of community development interventions [9].

Remote communities have the poorest access to health services generally [10]. Local availability of basic care, including mental health services, is critical to ‘closing the gap’ in health outcomes. The practical considerations of applying interventions locally also have implications for the way in which these services are evaluated. Evaluation should be seen as an important and necessary aspect of service provision to promote the integrity, value, and sustainability of an intervention. It is also axiomatic, however, that research in Indigenous Australian communities should be of value to the people who participate in the research [11,12]. Research of this kind is important in a broader sense as well because there is limited information available regarding the implementation and evaluation of interventions in Indigenous Australian settings [9]. Emerging evidence indicates that cultural adaptations to interventions improve outcomes, however, further work is called for to explore the ways in which interventions should be most effectively tailored to context [13].

In 2006 the Central Australian Division of Primary Health Care (CADPHC) received government funding to improve access to psychological services. At that time, a remote Indigenous community of more than 500 people was experiencing significant social and interpersonal problems including family violence and suicide. A request was made from community members to establish a social and emotional wellbeing service (SEWBS) in the community. The Health Service of the community supported the proposal and further requested: that community members be engaged in the new project; that male and female counsellors be provided, and; that a focus on community education, prevention, and early intervention as well as counselling be included. These requests are entirely consistent with policy recommendations outlined in *Ways Forward: National Aboriginal and Torres Strait Islander Mental Health Policy. National Consultancy Report.*[14] which states among other things that: mental health services provided for Aboriginal people must be developed in response to identified needs and be provided by Aboriginal organisations wherever possible; responsibility for programs and services must rest with Aboriginal people, and; culturally valid understandings must shape the provision of services (p. 19).

Since the commencement of SEWBS, quarterly reports have been produced and these, as well as anecdotal evidence, suggest that it has been a significant innovation for the community. One important and very tangible result has been a reduction in the numbers of episodes of self-harm, attempted suicides, and completed suicides occurring in the community (this is explained further with quotes from participants in the results section). After the service had been operating for more than three years, therefore, there were indications that it was a successful response to community problems. There was an expressed need, however, by members of the local health board to evaluate the service more formally so that evidence-based decisions could be made about the future direction of SEWBS and to ensure that community priorities continue to be addressed. Furthermore, it was also felt that lessons learned about the processes involved in establishing this service could be useful to people in other remote communities seeking to address similar problems.

A crucial consideration from the beginning of the research was a distinction between important processes and effective programs. It has been suggested that, due to the heterogenous nature of remote Indigenous communities it will be far more fruitful to identify processes – such as responsivity to community needs – that are important in promoting wellbeing than highlighting particular programs that have been effective in different communities [9,15]. This consideration was particularly important given that those people within the community who were seeking to have the SEWBS evaluated wanted the evaluation to provide useful information to people in other communities who might be interested in addressing similar problems. It is now recognised that an activity that is appropriate and effective for one community – a horse trek for example – may be inappropriate and ineffective for a different community. Whilst particular programs, therefore, may not be transportable from one community to another, important processes should be.

The Mpwelarre Health Aboriginal Corporation is the local health board comprising community members as well as health centre staff such as the general practitioner and the practice manager. The board is chaired by a local member of the community. The Corporation gave their permission and endorsement for the evaluation. The central research question was “What has been the impact of the SEWBS?”.

## Methods

### Design

The design of the research was a cross-sectional qualitative study. It was decided that the most appropriate method for answering the research question would be to conduct semi-structured interviews with a range of people involved in the SEWBS. To conduct the interviews the principal researcher recruited a research assistant who was experienced in conducting qualitative interviews. The research assistant was a non Indigenous female with doctoral qualifications and a background in nursing and mental health.

To help ensure free and informed consent a two stage recruitment process was adopted. First, the Lead Counsellor in the SEWBS generated a list of names of people who would be appropriate to participate in the research. The Lead Counsellor then contacted these people to inform them of the research and to seek their consent to be contacted by the researchers. Next, the Lead Counsellor provided the researchers with this list and, from this list, participants were recruited to the study. The Lead Counsellor was not aware of who agreed and who did not agree to participate. The initial list contained 40 names of whom 37 consented to be contacted by the researchers. Of these 37 potential participants, 21 people were interviewed. The remaining 16 people either could not be contacted in the time frame of the research or did not wish to be interviewed.

Interviews were conducted individually (with the exception of one interview where two people were interviewed together due to logistical reasons) and were planned to last between 30 and 60 minutes. Interviewees were offered $50.00 for participating in the interviews. Interviews were transcribed and all participants were offered the opportunity to review their transcripts. Topic guides were used to loosely structure the interviews (see Appendix).

### Setting

The SEWBS comprises a suite of activities conducted on an individual, family, and large group basis that are responsive to the needs of the community and its members. Activities include individual counselling, family therapy, narrative therapy, play therapy, sandplay, traditional healing, cultural activities such as men’s dancing, community engagement activities, and community education. Additionally, the four SEWBS service providers work with other health and allied health professionals from the Health Service and also from other organisations who visit the community.

While the suite of activities offered is impressive, for the reasons mentioned above, this research did not focus on evaluating specific activities. The activities that were developed in this context emerged from a combination of the skills of the service providers and the needs and interests of the community. There is no sense in which this particular array of activities is the “right” selection in order to improve the social and emotional wellbeing of Indigenous people in remote communities. For this reason, the focus of the research concerned the way in which the service generally was developed and maintained and not the effects of particular activities provided within the service.

At the time of the evaluation, the SEWBS comprised two Aboriginal Family Workers (AFWs; one male and one female) and two non Indigenous (one male and one female) counsellors. The two AFWs live in the community and the two non Indigenous counsellors spend between four and six nights a week living in the community. The female AFW is also a traditional healer which is an important aspect of the SEWBS given the fundamental connection between spirituality and health and wellbeing for Indigenous Australians [16].

### Procedure

A small number of common factors that help ensure research findings translate into practice have been identified [17]. In particular, focusing on outcomes, synthesising findings from a variety of sources, building strong relationships between researchers and stakeholders, and targetting multiple levels of change are particularly relevant for this evaluation. To help achieve this translation, the principal researcher spent approximately 12 months travelling to the community on a regular basis to develop and build relationships with community members as well as Health Clinic staff and SEWBS service providers. These visits included participating in activities such as camping overnight with people participating in the annual horse trek of the community. Knowledge from the discussions during these visits provided the principal researcher with an enhanced awareness of the functioning of the community, helped inform the design of the research, and promoted a greater understanding of the purpose of the research by members of the community and thus facilitated improved data access. The principal researcher is a non Indigenous clinical psychologist practitioner and researcher with experience in quantitative and qualitative methods.

The early stages of visiting the community involved clarifying the need for the evaluation and obtaining permission from the locally operated Health Board to conduct the research. After permission was granted from the Health Board the researcher obtained ethical approval through the Central Australian Human Research Ethics Committee to proceed with the research.

### Participants

People from a range of different groups were purposively sampled to ensure a broad spectrum of experiences would be included. The groups from which people were recruited along with the number of people in that group are provided in Table 1.

**Table 1:** The number of participants interviewed and their roles

**Role**	**Number of people contacted to be interviewed**	**Number of people interviewed**
Stakeholders	7	4
Referrers	4	3
Service Provider	4	4
Service Participants	14	7
Significant Others	8	3

Stakeholders were people who had been involved in the initiation and establishment of the service, Referrers were people who referred people to the service, Service Providers were the people who provided the service, Service Participants were people who participated in various activities of the service, and Significant Others were people who were partners or carers of people who had participated in activities of the service. Although some of the activities of the SEWBS involved working with children, only adult participants were recruited to the study. A total of 21 interviews were conducted and, of the people interviewed, 13 were male and 12 were Indigenous.

### Data analysis

Analysis was undertaken using Interpretative Phenomenological Analysis IPA; [18]. IPA was seen as an appropriate analytic strategy because the aim of IPA is “to explore in detail how participants are making sense of their personal and social world” [19], p. 53. After the interviews were transcribed, the analysis followed the IPA process. It is a cyclical, iterative process, with a constant revisiting of the transcripts to ensure that the superordinate themes that are generated directly relate to the shared experience of the participants [19].

Initially, all the transcripts were read and re-read and words or phrases that appeared important in terms of answering the research question were highlighted. These highlighted words and phrases were then collated into tables with each participant’s comments occupying one column. One table was collated for each group – Referrers, Stakeholders, and so on. Within these tables, each column was interrogated initially with similar comments and phrases being grouped together to create themes for each participant. These individual participant themes were then synthesised across participants to develop a coherent summary of the interviews. The original transcripts were frequently consulted during this phase of the analysis to ensure that the comments being synthesised retained their original meanings. Finally, the themes developed from the transcripts were grouped according to common concepts to form superordinate themes and subthemes. Table 2 presents the two superordinate themes with their subthemes.

**Table 2:** Superordinate themes and their subthemes

**Superordinate themes**	**The big picture**	**On the ground**	
Subthemes	Getting started	Personal struggles
	Organizational factors	Program activities
	Funding	Measuring outcomes
	The Future	Program results
	Operational problems		

## Results and discussion

In this section quotes from participants are included in the explanations of the subthemes provided below. Each quote is demarcated by an identifier in square brackets at the end of the quote. The identifiers are: S=Stakeholder; SPr=Service Provider; R=Referrer; SPa=Service Participant; SO=Significant Other. The subscripts with each identifier indicate a specific participant. Some participants commented that it was difficult to only provide comments from one perspective. For example, two Stakeholders have referred to the program and one Stakeholder had also been a Service Provider. Furthermore, some of the Service Participants were also Significant Others for other Service Participants and some of the Significant Others had participated in various activities of the SEWBS. Rather than being a disadvantage, it was felt that these enriched perspectives would enhance participants’ reports of the program even though they were asked to focus on the role for which they were being interviewed.

The participants’ views of the SEWBS were largely concordant with a general feeling that there had been a positive and significant impact on the community. Generally, the experiences described by the participants of the SEWBS could be grouped into two superordinate themes: The Big Picture, and; On the Ground. Interestingly, the reports of the participants did not clash in anyway and nor did they contradict each other. Some participants commented more about some aspects of the SEWBS than other participants. For example the Stakeholders and Service Providers, but not the Service Participants or Significant Others, discussed organisational aspects of the program such as funding arrangements and staffing. Additionally, some participants expressed ideas about improving the SEWBS but, generally, the participants were all very positive about the initiative.

The superordinate theme “The Big Picture” covered aspects of the SEWBS that included the organizational considerations involved in its establishment and ongoing operation. The superordinate theme “On the Ground” included themes that encompassed the operation of the SEWBS including the problems it addressed and the effects it had. Each of the subthemes is described below.

### The big picture

#### Getting started

Participants discussed the negotiation processes that were initially involved in bringing organizations together to develop a new way of working within the community. From the outset there was an awareness that developing the service within the community would take time. It was also acknowledged that luck and opportunism were involved as well as making use of existing relationships to recruit staff to the service.

*“We took care to ensure that everybody who needed to knew about it and, as I say, thought there was room for a new service. It was really just - obviously finding a suitable person to kick it off was always going to be difficult.”* [S_1_]

*“The relationships that people build with community members are very slow and fragile in getting them going … We all know that service providers who attempt to provide from* [a larger town] *to remote communities do only touch the surface because they, unfortunately, can only drive in, have a meeting and drive out. Community members don’t tend to work like that; they don’t want to work like that so they will turn towards people who they know, who are residential in the community, who they’re familiar with.”* [S_2_]

#### Organizational factors

The importance of organizational and systemic factors was emphasised by many participants. Different organizations were involved from the outset and this increased the complexity of such things as priorities, expectations, accountability, and reporting. There was also a sense that decisions were sometimes made by people who were not located in the community and were not always aware of the specific needs of the community.

*“… there were four players in this program and there were four programs, all with different accountability and all with very uncertain timelines, so a dreadful mess.”* [S_2_]

#### Funding

Participants described the difficulties that occurred by having funding for the program provided by different organizations. They used terms such as “messy” and “cobbled together”. In some ways, having different organizations involved, provided an opportunity to get more funding than might have otherwise been the case.

*“On a positive note we were able to get funding for additional positions and we did.”* [S_1_]

There were significant problems, however, with funding provided by different organizations with different expectations. One organization, for example, decided they would not provide ongoing funding. Sustainable funding, therefore, was a recurring problem and this was compounded by the sense that the work that would be helpful in a remote Indigenous community did not always fit into “white fella” funding and accountability structures.

*“often most of our white fella systems are really good at supporting us to get education or employment or whatever but they’re all white fella things. It’s about money or image and - whereas most of them want a sense of identity of who they are as Aboriginal people as well but there are very few opportunities that are funded to make that happen.”* [SPr_1_]

#### The future

An indication of the success of the service was provided by the way in which participants discussed ideas for expanding the service both within the community and also encouraging other communities to adopt similar services. Participants mentioned the need for capacity building within the community to increase the number of local people developing leadership roles and they also indicated a clear preference for community-based rather than fly-in fly-out services. They also identified areas yet to be investigated such as a cost benefit analysis.

*“… to include a cost benefit sort of analysis so we can sell it to government as a significant new initiative which has benefits across a whole range of areas rather than the alternative fly-in, fly-out white experts into places.”* [S_3_]

*“Like I said I’d just like to see more jobs available for people that are on the ground in the community and like I said, communities have got different ways of dealing with their communities and we’ve got different cultures and the best way is to work with your own culture and that’s where it’ll bring that standard back … just to respect and show honour.”* [SPr_2_]

*“The ideal would be to support other community workers to have that sort of role in the community and to really have an Indigenous response of support that they are really happy with and determine themselves.”* [SPr_3_]

#### Operational problems

Participants discussed various problems in establishing, maintaining, and promoting access to the service. There were problems with recruitment and also problems in the scope of the work required given the time available. Some participants had a sense that the demands of the position could leave the service providers feeling exhausted and overwhelmed. Also, communication barriers sometimes had to be negotiated if the service provider and the person accessing the service were from different skin groups or if there were differences in their abilities with the use of English and Indigenous languages.

*“I’d like to see more … feet on the ground because I feel that sometimes it’s just manic and … a very short amount of time to … work in.”* [R_1_]

*“… some people won’t go … it’s because they can’t because of cultural reasons, because of the skin group, that they’re that side but the others aren’t covered.”* [SO_1_]

### On the ground

#### Personal struggles

Participants described a range of different problems that the service addressed. Violence, suicide, and self-harm were the most serious problems, however, problems with alcohol and other drugs, as well as a lack of opportunities and control, conflicts of life, trauma, and relationship difficulties were among some of the other problems mentioned. There was an acknowledgement that the problems were complex and occurred at different levels.

*“Yeah mainly my kids put a lot of pressure on me. We used to live all squashed up in one house and I had my sister-in-law with us and her nephews and nieces and my two kids, all squashed up – and my partner – and that was all making it hard to cope.”* [SPa_1_]

*“…he got drunk and fight with families, with me … He was being abusive.”* [SPa_2_]

*“… we worked out that over a two year period 30 people had either completed or attempted suicide over a two year period, out of a population of 600.”* [S_1_]

These suicides or suicide attempts all occurred prior to the introduction of the service. Significantly, some participants reported that there has been only one suicide since the program started over three years ago.

*“Well in this community there was a lot of alcohol issues and drugs and suicide of young people, with their relationships and stuff.”* [SPr_2_]

#### Program activities

The importance of working flexibly was evident in the way the participants described the programs within the service. Both individual as well as community development work was undertaken. There was a sense of teamwork among the providers of the SEWBS, colleagues in the clinic, and other local groups. Having someone to talk to who was trusted seemed to be important. The various programs that people could engage in were also beneficial.

*“… the activities of providing meaningful employment and meaningful activities for people is certainly very much a part of trying to improve the general mental health of people, especially the men, and that helping them to think about enterprises and things that they might do should be part of this program and that the community development aspect is fundamental to it.”* [S_1_]

*“I think because a lot of us Aboriginal people are pretty shy and don’t really like talking to other family members so it’s good to talk with someone that you don’t know.”* [SPa_3_]

*“Yeah we definitely work as a team … If I’m having difficulties getting my ideas across to a family or family group or person I’ll talk to him* [one of the service providers] *and … we bounce ideas off.”* [R_1_]

*“Talking about it made me think, like it sort of straightens you out and – like talking and planning ahead too …”* [SPa_4_]

#### Measuring outcomes

Participants communicated an awareness of the importance of accountability in terms of being able to assess the impact of the service. At the same time, however, there was an acknowledgement of the difficulties of measuring the kind of different work that was required in a remote Indigenous community.

*“There’s lots of things going on and to actually show that what you’ve done has actually improved the lives of these people is always a bit trickier …”* [S_4_]

#### Program results

Despite acknowledging the difficulties in measuring change and assessing impact, participants spoke about a range of different activities and strategies that were provided and how the assistance that was offered enabled them to cope better with difficult circumstances. Most participants described benefits they had noticed at both a community and individual or family level. They described tangible changes such as reductions in smoking, alcohol, and self-harm as well as less concrete changes such as increased engagement and a sense of value.

*“I think the social, emotional health of our community speaks for the value of this program, that in the several years that the program has been running the self-harm aspects of health service delivery have, from our point of view, really dramatically reduced.”* [S_2_]

*“… would come and see us and talk about strategies of how we could work together and solve issues and be happy again … helped us a lot. Things are much better now and we’re working through it together.”* [SPa_3_]

*“I know they haven’t been smoking much, not when they’re back here now. I’ve noticed they’re not worrying about drugs anymore, just worrying about their kids and at the weekend they was cleaning up the house and doing the washing …”* [SO_2_]

These results indicate that the participants of this study feel that the SEWBS has been an effective response to significant community problems. The combination of preventative work through community development activities as well as responding to crisis situations through the availability of personnel who were able to provide opportunities for community members to openly talk about their problems was a particular strength identified by participants.

The reported effectiveness of the program is all the more remarkable given the significant hurdles that needed to be overcome as well as the unanticipated obstacles that continue to be negotiated. The provision of funding by different funding bodies with no clear accountability or decision-making pathways was a serious problem. This was compounded by the fact that decisions were being made by non Indigenous people who had little understanding of the conditions and characteristics of remote Indigenous communities. One of the consequences of this was a feeling by the SEWBS service providers that what the funders wanted to fund was a service that fixed problems based on a deficit model whereas what was needed in the community was a holistic approach that was more strengths-based.

Being able to measure or assess the impact of the program was another tension. The reporting requirements of the funding bodies needed to be balanced against the requirements of the role of service provider. It seemed evident that many of the ways of evaluating services that might be adopted in urban centres would not be appropriate in the context of a remote Indigenous community. For example, because of the heterogenous nature of remote communities comparative research is problematic. Also, designs such as randomised controlled trials (RCTs) which are often considered the gold standard in service evaluation are frequently impractical in remote communities due to, among other things, small sample sizes, as well as the questions of interest being unsuitable for an RCT design. Finally, the time frame within which it will be reasonable to expect change to have occurred will be longer in a remote community compared with an urban setting. Despite these caveats it seemed clear that important changes had occurred in the community so, at least in principle, it should be possible to capture these changes in some way.

## Conclusions

Providing a localised response to significant social and interpersonal problems in a remote Indigenous community appears to have resulted in a reduction in these problems. Issues of effectiveness, access, and sustainability are fundamental if outcomes are to be maintained so that the people living in these communities are able to live lives they have reason to value. Evaluations such as the one described in this paper are important for promoting a clearer understanding of the critical processes and issues involved in securing lasting change.

From this evaluation a number of conclusions can be made. The conclusions that emerged can be loosely grouped into “recommendations” and “priorities for future research and development”. The conclusions are outlined in Table 3 and explained in detail below. The strength of these conclusions needs to be considered within the context of this research being one qualitative study of one social and emotional wellbeing service in one community. Nevertheless, given the nature of the conclusions, they are likely to be broadly applicable.

**Table 3:** Recommendations and priorities for future research and development

**Recommendations**	**Priorities for future research and development**
1. Appropriate staffing of the program is critical	1. Investigate ways of localising decision making
2. Involve residents from the community in decision-making about programs in the community	2. Conduct cost/benefit analyses
3. Identify priority outcomes and how they will be evaluated	3. Identify ideal staff/community ratios
	4. Develop training models of local relevance including teaching local languages
	5. Clarify recruitment priorities in terms of connectedness and culture
	6. Improve flexibility around position descriptions and qualifications

### Recommendations

To ensure the sustainability of this program a number of recommendations can be offered.

#### Appropriate staffing

Appropriate staffing of the program is critical. While recruitment will clearly be important, it is vital that once staff are recruited that they receive adequate supervision, support, and training. It is important that workloads are managed to prevent burn-out and that conditions such as wages and accommodation are negotiated in an open and transparent way. It is also essential that funders and policy-makers develop a more refined understanding of the complexities of living in a remote Indigenous community. Remote settings have particular implications for health service delivery and providing health services to Indigenous people has different implications. The effective and sustained delivery of a health service in a remote Indigenous community, therefore, will require sophisticated planning. Even simple things such as the time frame within which it is realistic to expect change will be different in a remote Indigenous community compared to an urban setting.

#### Involve community residents

To maximize the most appropriate and efficient use of resources it is also recommended that people from the community be involved in decision-making about services in the community. Resident services appear to be preferred to fly-in fly-out services but perhaps more important than the way the service is actually delivered is the extent to which community members have been able to determine the services that are most appropriate for their local context.

#### Identify priorities

Measurement issues were a concern to a number of participants and while this is difficult it should not be seen as an insurmountable problem. In fact, the same ingenuity and creativity that has been used to develop and provide services within the community could be used to find innovative ways of evaluating their impact. Participants reported reductions in the use of alcohol and other drugs. It should be possible to quantify these reductions. Participants also reported increased engagement in community activities. If attendance is considered to be a sign of engagement then, once again, documenting changes in numbers attending particular community activities should be relatively straightforward.

### Priorities for future research and development

Perhaps one of the most impressive aspects of the SEWBS was the willingness of the service providers to respond to community needs and to change and adapt the programs as circumstances required. Using this approach some significant learnings occurred which provide useful hints for where it might be beneficial to focus future research and development activity.

#### Localise decision making

It seems very clear that services work more effectively and efficiently when local decision makers are involved. The processes developed in this community could be introduced to other communities to assess their impact on family and community relationships. The establishment of a committee with representation from the community as well as the funding body that could serve as the link between the community and the funders would be critical. This committee could be the focal point for establishing community priorities as well as the desirable outcomes to be evaluated. They could also serve a coordinating function through which outside services could be managed to ensure that duplication of services did not occur and that the services were being provided according to community need not funding priorities or some other standard.

#### Cost/benefit analyses and staff/community ratios

Introducing this communities’ process of holistic community engagement to other communities would provide an opportunity to conduct large scale and more rigorous research. It would be useful, for example, to include a cost/benefit analysis and also to investigate issues such as the most desirable ratio for staff to community. It is likely that the ratio of health worker to community on a remote Indigenous community will be different and more nuanced than the ratio of health worker to community in an urban or metropolitan setting. It would be important for example to ensure a gender balance as well as appropriate coverage of skin groups and other family groupings. Future research could also consider the issue of capacity building, the development of leadership within the community, and the ultimate sustainability of the program.

#### Relevant training models recognising connectedness and culture

Another important focus for future research might be the value of local knowledge including fluency in local languages. Is local knowledge and local connectedness more important than, for example, Indigeneity? Would it be more important to recruit a person who spoke Arrernte (a Central Australian language) and was well connected to local family groups than it would be to recruit an Indigenous person from interstate who was better qualified and had more experience? If support was provided to new staff to learn the language of the community (such as Walpiri or Pintupi Luritja – other Central Australian languages) would that improve retention, enhance cultural knowledge, and increase job satisfaction?

#### Position descriptions and qualifications

The issue of qualifications will also be important to consider in future research and development programs. Could there be a different way of recognizing skills and knowledge than current qualifications and job descriptions allow? Research looking at different staffing models might persuade funding bodies to expand their criteria for suitable position descriptions to accommodate the demands at a local level.

According to the views of the participants of this study, a Central Australian remote Indigenous community has benefitted from a flexible and multifaceted response to significant community problems. From the experiences of people in the community who were involved in the development and delivery of the service it is possible to extract key learnings with regard to service effectiveness, access, and sustainability. Perhaps one of the cornerstones to the success of the service has been the commitment of the people providing the service to involve the community in the direction and scope of the service. If the decision and policy makers who fund these kinds of services could learn from this and find ways of promoting legitimate self-determination and control within remote Indigenous communities then perhaps services such as the SEWBS would be even more effective and widespread.

## Appendix

### Topic guides used in the interviews

#### stakeholders and service providers

1. Establishment of the SEWB Team

a. Motivation?

b. Hurdles, problems, barriers?

c. How were they overcome?

2. Progress of the SEWBP

a. What were things like initially?

b. What are they like now?

c. Pace of progress?

d. Tracking of progress, evaluation procedures, accountability?

3. Benefits of the SEWBP

a. What has been noticed?

b. Who has noticed it?

c. Has it been worthwhile?

4. Future ways of working and activities for the SEWBP team

a. What next?

b. What would you recommend to other communities?

#### Referrers

1. Knowledge of the what the SEWBP team does and how it works

a. How did you learn about it?

b. What do you know of it?

c. Has it changed over time?

2. Use of the SEWBP

a. How much do you use it?

b. What do you use it for?

c. Are there any things you wouldn’t use it for?

3. Perspective of the SEWBP

a. What has your experience of the program been?

b. What benefits have you noticed?

c. Could anything be different?

4. Advice for the SEWBP team

a. Recommendations?

#### Service participant

1. Before the SEWBP activities

a. What was happening?

b. For how long?

c. How did you see yourself?

d. Had you tried anything else?

2. During the SEWBP activities

a. What happened?

b. For how long?

c. What do you remember?

3. After the SEWBP activities

a. How are things now?

b. What’s different?

c. Any changes still to make?

#### Significant other

1. Before the SEWBP activities

a. What was X like?

b. For how long?

c. How did you feel about it?

2. During the SEWBP activities

a. What happened?

b. Was there any change?

c. How did you know?

d. What did you notice?

3. After the SEWBP activities

a. How are things now?

b. What’s different?

c. Any changes still to make?

## Competing interests

The author had no competing interests in the conduct of this research or the preparation of this manuscript.

## Authors’ contribution

TAC is the sole author of this publication. He was responsible for all stages of the research including research design, obtaining ethics approval, supervising data collection, data analysis, and preparation of the manuscript for publication.

## Pre-publication history

The pre-publication history for this paper can be accessed here:

http://www.biomedcentral.com/1472-6963/13/80/prepub
